# The PID Principles of Care: Where Are We Now? A Global Status Report Based on the PID Life Index

**DOI:** 10.3389/fimmu.2021.780140

**Published:** 2021-11-18

**Authors:** Julia Nordin, Leire Solís, Johan Prévot, Nizar Mahlaoui, Helen Chapel, Silvia Sánchez-Ramón, Adli Ali, John W. Seymour, Martine Pergent

**Affiliations:** ^1^ The International Patient Organisation for Primary Immunodeficiencies, Downderry, United Kingdom; ^2^ Pediatric Immunology-Hematology and Rheumatology Unit, Necker Children’s University Hospital, Assistance Publique-Hôpitaux de Paris (AP-HP), Paris, France; ^3^ French National Reference Center for Primary Immune Deficiencies (CEREDIH), Necker Children’s University Hospital, Assistance Publique-Hôpitaux de Paris (AP-HP), Paris, France; ^4^ Nuffield Department of Medicine, University of Oxford, Oxford, United Kingdom; ^5^ Department of Clinical Immunology, Instituto de Medicina del Laboratorio (IML) and Instituto de Investigación Clínico San Carlos (IdISSC), Hospital Clínico San Carlos, Madrid, Spain; ^6^ Department of Immunology, ENT and Ophthalmology, Complutense University School of Medicine, Madrid, Spain; ^7^ Clinical Immunology Unit, Department of Paediatrics, Faculty of Medicine, Universiti Kebangsaan Malaysia Medical Centre, Kuala Lumpur, Malaysia; ^8^ Institute of IR4.0, Universiti Kebangsaan Malaysia, Bangi, Malaysia; ^9^ Department of Counseling and Student Personnel, Minnesota State University, Mankato, MN, United States

**Keywords:** primary immunodeficiency, global PID data, diagnosis, treatment, universal health care, specialised centres, PID patient organisations, registries

## Abstract

A global gold standard framework for primary immunodeficiency (PID) care, structured around six principles, was published in 2014. To measure the implementation status of these principles IPOPI developed the PID Life Index in 2020, an interactive tool aggregating national PID data. This development was combined with a revision of the principles to consider advances in the field of health and science as well as political developments since 2014. The revision resulted in the following six principles: PID diagnosis, treatments, universal health coverage, specialised centres, national patient organisations and registries for PIDs. A questionnaire corresponding to these principles was sent out to IPOPI’s national member organisations and to countries in which IPOPI had medical contacts, and data was gathered from 60 countries. The data demonstrates that, regardless of global scientific progress on PIDs with a growing number of diagnostic tools and better treatment options becoming available, the accessibility and affordability of these remains uneven throughout the world. It is not only visible between regions, but also between countries within the same region. One of the most urgent needs is medical education. In countries without immunologists, patients with PID suffer the risk of remaining undiagnosed or misdiagnosed, resulting in health implications or even death. Many countries also lack the infrastructure needed to carry out more advanced diagnostic tests and perform treatments such as hematopoietic stem cell transplantation or gene therapy. The incapacity to secure appropriate diagnosis and treatments affects the PID environment negatively in these countries. Availability and affordability also remain key issues, as diagnosis and treatments require coverage/reimbursement to ensure that patients with PID can access them in practice, not only in theory. This is still not the case in many countries of the world according to the PID Life Index. Although some countries do perform better than others, to date no country has fully implemented the PID principles of care, confirming the long way ahead to ensure an optimal environment for patients with PID in every country.

## 1 Introduction

Primary immunodeficiencies (PIDs) are a large and growing group of over 450 (1) different disorders caused when components of the immune system are not working properly. Whilst PIDs are generally recognised as rare disorders, some are more common than others and together they represent an important group of people whose lives are profoundly impacted by their condition. PIDs are related to inherited defects of the immune system (genetic or epigenetic). A normally functioning immune system maintains the integrity of the body, by fighting off infections by germs such as bacteria, viruses, fungi, and protozoa and tumour cells, and by keeping the internal homeostatic equilibrium. Because of their impaired immune system, people with PIDs are more prone to infections, autoimmune diseases and dysregulated inflammation than others. When PIDs are left underdiagnosed or are misdiagnosed, the defective immune system leads to illness, disability, permanent organ damage and even death (2).

On a global level, the field of PIDs has been developing vastly during the past decades (3) with new genes being discovered and precision medicine further explored. This has noticeable happened faster in some parts of the world than in others. Parallel to this and since 1992, the International Patient Organisation for Primary Immunodeficiencies (IPOPI) has advocated to increase awareness and to improve access to early diagnosis and optimal treatments for PID patients worldwide. One important step in this process was to establish a global gold standard framework for PID care, in the shape of six PID Principles of Care published in 2014 by a global multidisciplinary group of PID experts (2). In 2020, this was followed by the development of the PID Life Index, a global index measuring the implementation of the PID Principles of Care in countries all over the world. In the development process of the PID Life Index, the PID Principles of Care were updated to apply to the current PID global environment more accurately (see *Methods*), and thus the principles included in the PID Life Index are: 1) Availability of diagnosis; 2) Availability of treatment; 3) Universal Health Coverage; 4) National Specialised centres; 5) National Patient Organisations; 6) National registries. The PID Principles of Care implementation survey was the first of its scale, extending also outside the IPOPI membership. This article provides an overview of the general status of the Principles of Care on a global scale, paving the way for the possibility to present more detailed and tailored results in future publications. This publication presents the results of the data gathered in 2020 and the first half of 2021 from 60 countries.

## 2 Methods

The PID Life Index development has been described (article pending publication). The Index is a result of a reflection concerning the PID principles of care as published in 2014 (2), regarding how to assess the level of implementation of these principles as well as how to create a tool that captures the status of the PID healthcare environment in each country. To start, the 6 PID principles of care were reviewed to consider advances in the field of health and science, as well as political developments that had occurred after their initial publication in 2014. For this IPOPI engaged the expertise of 3 dedicated medical experts in the field of PIDs, as well as PID patient representatives from national member organisations representing all PIDs. Based on the revision of the original principles, the list of principles was updated, and a new principle was added. From the 2014 landmark publication, the only new principle is universal health coverage, which replaced “managing PID diagnosis and care in all countries” and links the Index with the World Health Organisations initiative on Universal Health Coverage. The six established principles were: diagnosis, treatment, universal health coverage, specialised centres, national patient organisations and registries.

The principles were then structured and measured through a series of criteria. Consideration was given to the different criteria used to define the principles and the same consideration was given to the 6 principles aggregated within an index. This PID Life Index, encompassing the principles and the criteria, is displayed in a friendly data visualisation interface, making it possible for the user to prioritise one or some over the others.

Then, a questionnaire was built to provide the PID Life Index with national data gathered from IPOPI’s national patient organisations and from countries in which IPOPI had medical contacts. The questionnaire was tested in a limited number of countries in a pilot phase that helped to improve and validate the questionnaire. Once finalised, the questionnaire was sent to all IPOPI national patient organisations and a set of additional countries where IPOPI had medical contacts. The national member organisations were asked to respond to the questionnaire in collaboration with their medical advisors, and the information received was provided by IPOPI’s counterparts to the best of their knowledge. The result is a first in terms of the scale compilation of data on PID globally.

This article describes and discusses the results gathered from 60 countries in 2020 and the first half of 2021. The information has been divided into principles to assess how countries perform in each of them.

## 3 Results

### 3.1 Principle 1: Diagnosis of PIDs in the World

The principle of any PID diagnosis is based on 4 main categories: biological tests, genetic diagnosis, prenatal tests and newborn screening. A country with these 4 categories available for its citizens would be equipped to provide the best standard of diagnosis. According to the PID Life Index, 53 out of 54 countries responding to this principle provide at least one type of diagnostic test to its citizens. The most frequent test is biological diagnosis [some of which are recognised as essential tests by the World Health Organisation (WHO) (4)] followed by genetic diagnosis, prenatal testing and newborn screening. According to the data, biological diagnosis is fully available in 31 surveyed countries, genetic diagnosis in 26 countries and prenatal diagnosis in 21 countries. There are only a few countries in the world that offer the full range of diagnostics tools to their citizens (Germany, Sweden, United States of America and Iceland). The main reason for this being that newborn screening for Severe Combined Immunodeficiency (SCID), a paediatric emergency for which there is a treatment and, in many cases, a curative treatment, remains to be implemented in many countries at a national level.

**Figure 1:**
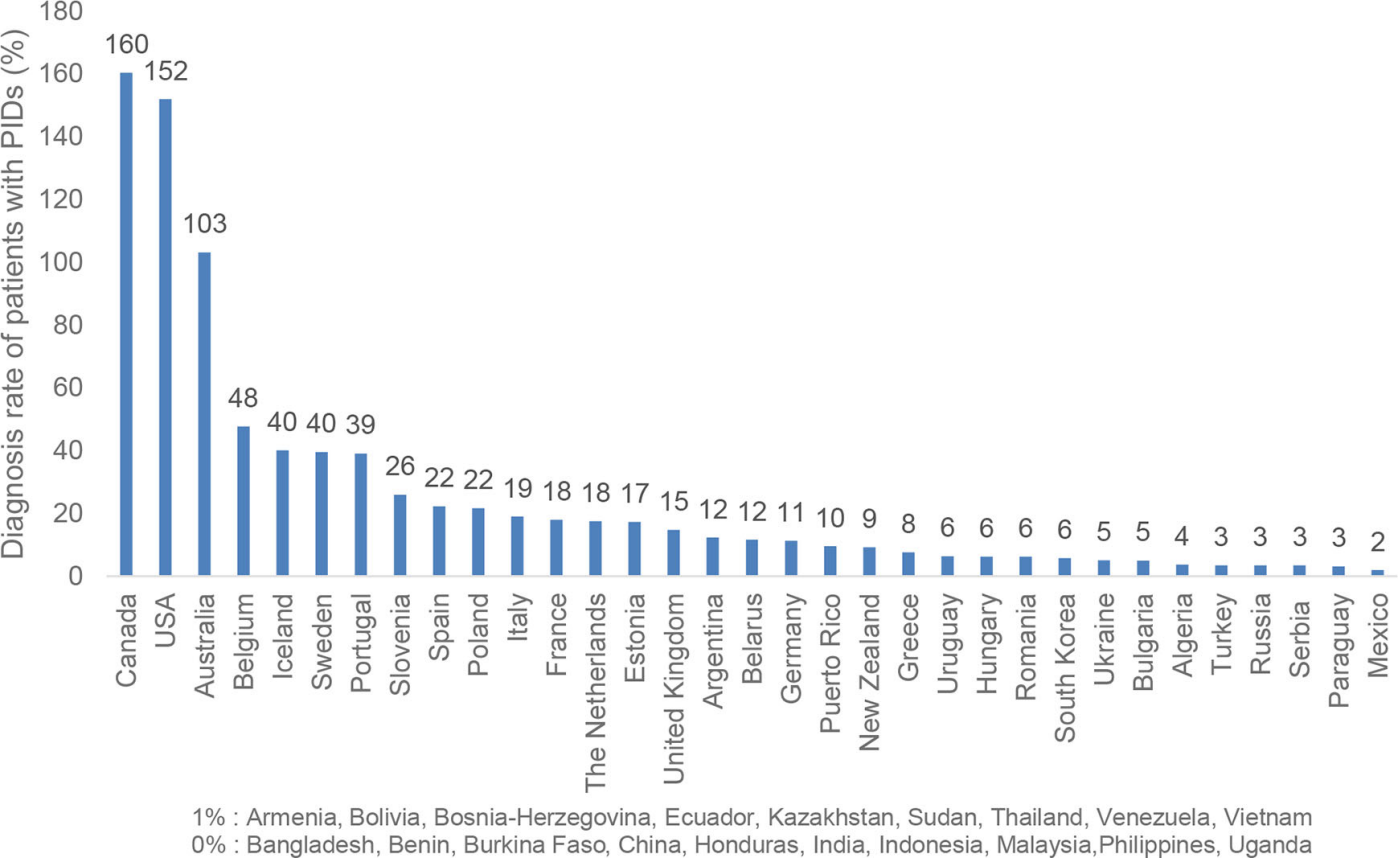
Diagnosis Rate of PIDs per National Population* (n=52). * Countries such as Canada, the United States and Australia calculate the number of patients with PIDs based on higher prevalence that includes even asymptomatic antibody deficiencies (IgA) which is not the case in the rest of the world. This explains the score above 100%.

The PID Life Index also investigates the diagnosis rate of the different countries (**Figure 1**), displaying data gathered between March 2020 and June 2021. This rate has been calculated based on the known number of patients and the theoretical number of PID patients in the country (based on a prevalence of 1 in 2,000 inhabitants, all PIDs included). When submitting the data, the responding countries have indicated where the data comes from, majority of the examples being from a registry, from a few centres, or from an estimate based on prevalence like the case with Canada, the United States and Australia (**Figure 1**).

Although it has generally been accepted that PIDs are under-diagnosed and under-reported, the PID prevalence rate is still discussed (5). This brings additional challenges to the measurement of this principle and explains why Canada, the United States and Australia have reported differently (**Figure 1**). It is also important to bring attention to the low estimated diagnosis rate in densely populated countries such as India and China. In this case their estimated low diagnosis rates are the result of the lack of national registries in these countries, which has led to them reporting a low number of known patients, resulting in very low diagnosis rates compared to the size of their populations.

#### 3.1.1 SCID Newborn Screening

**Figure 2:**
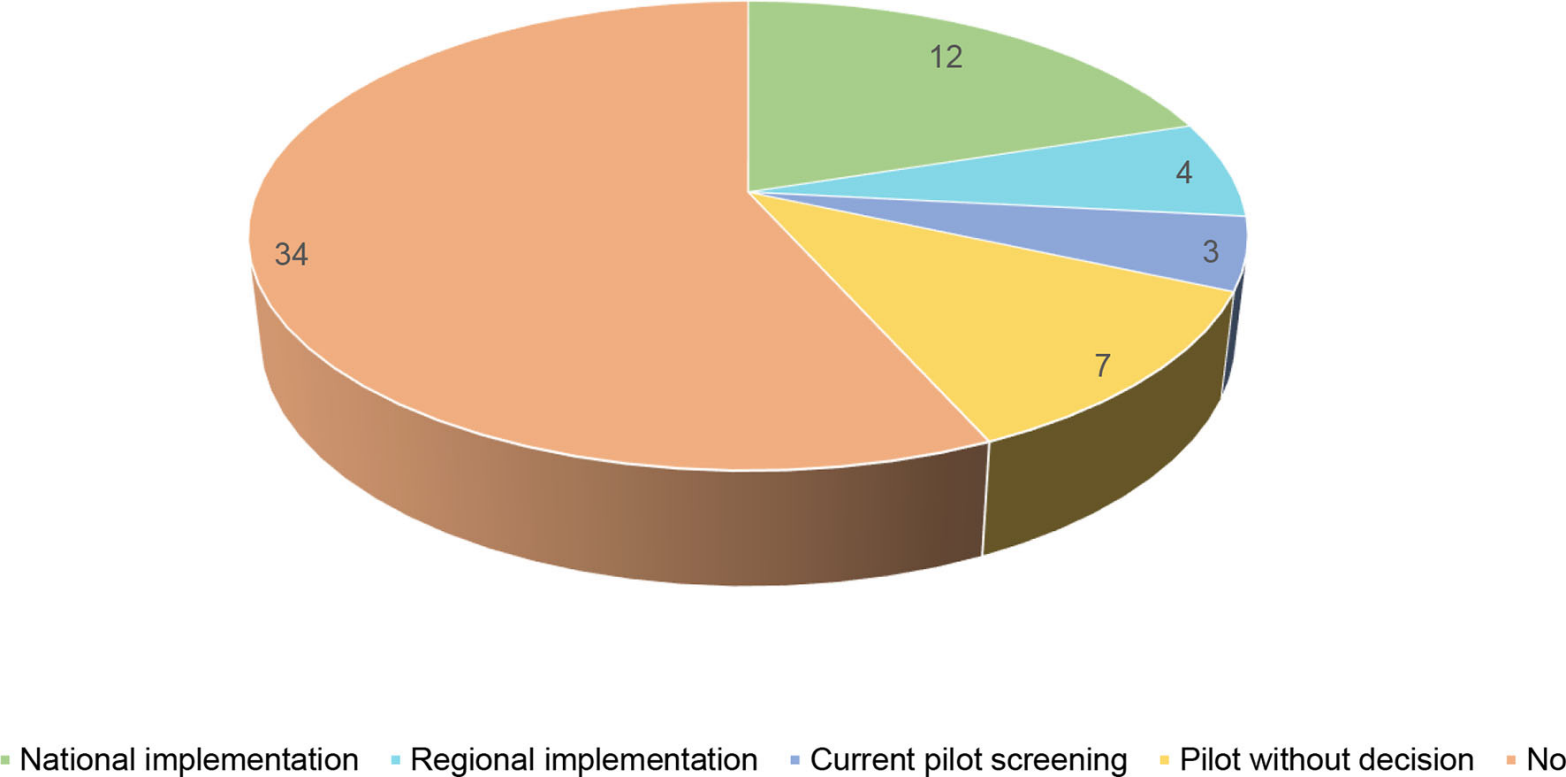
Newborn screening for SCID in the world (n = 60).

Newborn screening for Severe Combined Immunodeficiency is a lifesaving tool that allows for a swift diagnosis of the disease and timely treatment, normally through bone marrow transplantation or through hematopoietic stem cell transplantation (HSCT) and in some cases, gene therapy (6). For many years, detection of SCID cases was mainly done in families that had already lost a previous child due to SCID. In 2010, the United States was the first country to recommend the implementation of newborn screening for this disease at a national level, becoming a reality for all the states’ screening programmes in 2018. Since then, a growing number of countries have implemented newborn screening for SCID on national or regional level, but the majority of the countries still do not offer this screening to all newborns (**Figure 2**).

### 3.2 Principle 2: Availability of Treatments for PID Patients Worldwide

Depending on the nature of their gene defect, PID patients will need access to different kinds of treatments. These treatments can be supportive (e.g., immunoglobulin replacement therapy, anti-infectious therapies) or curative (HSCT, gene therapy or thymic transplant). In the PID Life Index therapies have been considered available if they have received a market authorisation and are present or can be accessed by patients with PIDs in the country, irrespective of whether they need to pay for them or if they are covered by the reimbursement health scheme or their national health system.

#### 3.2.1 Availability of Anti-Infectious Therapies

Anti-infectious therapies are used to tackle infections in PID patients and include antibiotics, antivirals, antifungal and antiparasitic medicines. These therapies are mainly given to many patients with PIDs as a prophylactic measure to prevent infections and on clinical diagnosis to tackle severe infections. Most of the anti-infectious therapies used to treat patients with PIDs are available in the countries included in the PID Life Index. However, some countries, such as Sudan, India, Venezuela, Ukraine and Mexico, report having only partial access to these therapies.

Anti-infectious therapies have a very specific mechanism of action against particular pathogens and, for patients with antibody deficiencies, cannot be used, as an alternative to immunoglobulin replacement therapy (IgRT).

#### 3.2.2 Immunoglobulins

Over 50% of affected PID patients suffer from antibody deficiencies (7). The main treatment for these forms of PID consists in the replacement of the missing antibodies *via* immunoglobulin replacement therapy (Ig). The treatment can be administered intravenously (IVIg) or subcutaneously (SCIg or facilitated fSCIg) at regular intervals and aims at increasing serum IgG trough levels to physiologic concentrations to protect against bacterial and viral infection (8).

According to the Principles of Care, “All countries and immunodeficiency centres should have access to a wide spectrum of Ig products, to provide optimal treatment for all immunodeficient patients” (2 p. 6). As this is a lifelong treatment and a biological medicine subject to differences in tolerance from one patient to another, it is vital to offer PID patients a wide range of products and administration routes to find what suits them best and ensure good quality of life. Furthermore, the WHO Essential Medicines List states immunoglobulins as essential therapies in the treatment of PIDs in adults and paediatric populations (9, 10) and therefore as a medicinal product that is considered to be most effective and safe as to meet the most important needs of national health systems. Numerous other international organisations such as the Asian-Pacific Economic Development Forum (APEC) and the Council of Europe have stated the essential nature of these therapies and the need for continued access to them by patients with PIDs (11, 12). Despite this, the availability of this life-saving treatment is still poor, or even non-existent, in many parts of the world. In countries where this is not available, PID patients are being denied access to the lifesaving and optimal treatment that they should receive, endangering their lives and health.

**Figure 3:**
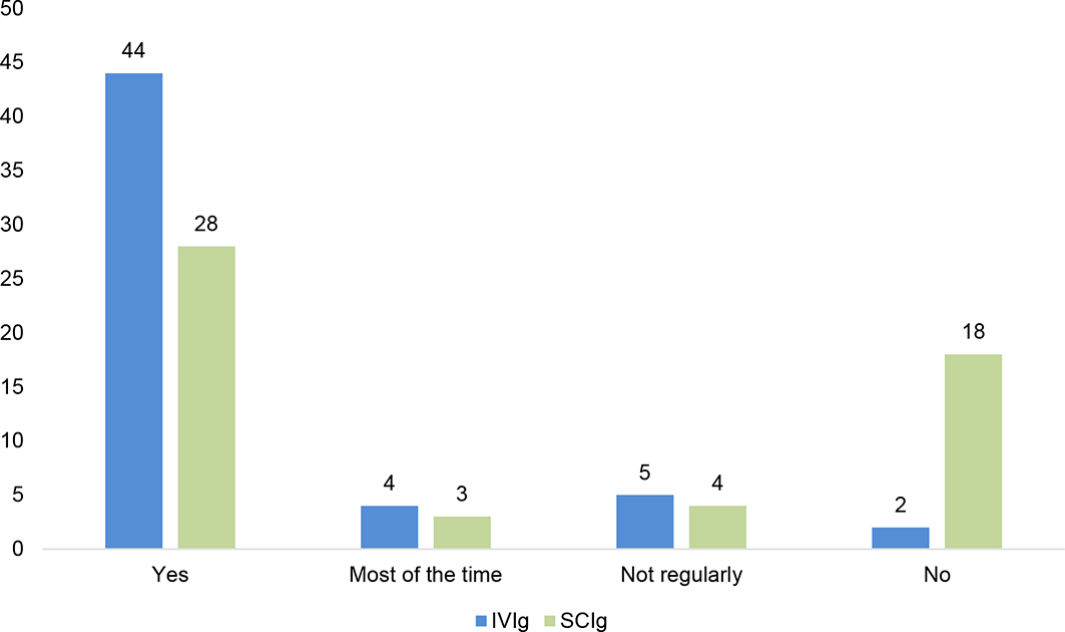
IVIg (n=55) and SCIg (n=53) availability at national level.

Compared to the availability of anti-infectious therapies, few countries report having access to the full range of Igs (**Figure 3**), mainly the US and some countries in the European Union. In terms of administration routes, IVIg is the option available in most countries: 44 out of 55 participating countries report having access. IVIg was the first successful product to be developed and is mainly provided in a hospital setting to patients. It has acquired a recognition that is unfortunately not yet present for SCIg or fSCIg in all countries. Still, out of the 55 respondents, 7 report having no access to IVIg therapy at all or regularly, indicating in most of the cases that patients in those countries cannot rely on regular and stable access to Ig therapy. SCIg introduced a great potential for patients having problems with their vein access or desiring to receive their infusion at home. Despite its international recognition by the WHO and other inter-institutional organisations, the subcutaneous route (SCIg and fSCIg) is still not widely available, only in 28 out of 53 countries.

**Figure 3** shows the long route ahead for PID patient organisations and PID specialists on the road of advocacy for accessing all routes of administration, to ensure a patient-centred approach to individualised care for PIDs.

#### 3.2.3 Curative Treatments

**Figure 4:**
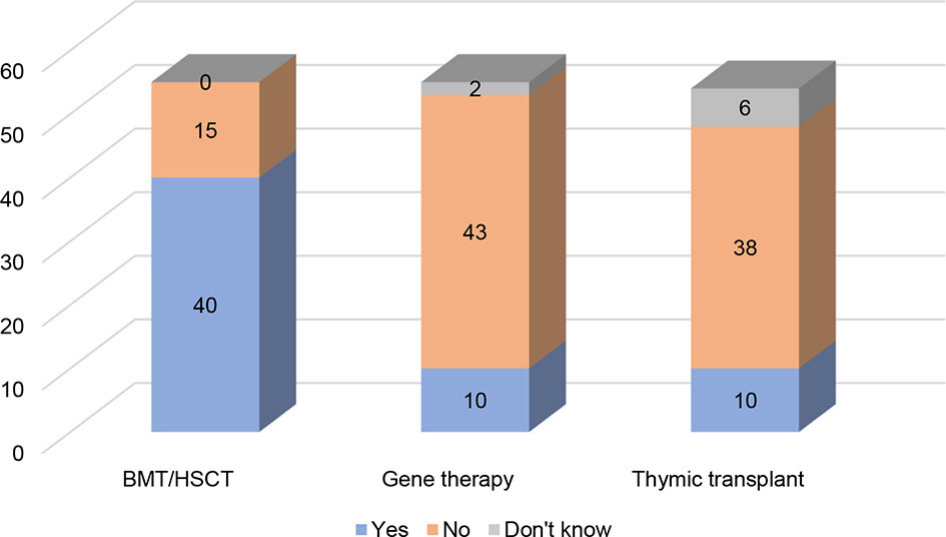
Availability of HSCT, GT, and thymic transplant (n= 55, 55, 54).

Some PIDs can be diagnosed and treated with curative interventions, such as hematopoietic stem cell transplantation (HSCT) either by bone marrow transplantation (BMT), peripheral blood stem cell transplantation (PBSCT) or umbilical cord blood transplantation (UCBT) depending on the source of the stem cells, gene therapy or thymic transplant. Since PIDs are rare and often have unique aspects intrinsic to each PID and the patient, it is hard to define a universal transplant regimen (13). Despite this difficulty they have proven to be effective treatment options for some PIDs, examples including SCIDs, Wiskott-Aldrich syndrome (WAS) and X-linked hyper immunoglobulin M syndrome (XHIM) (14). For some, such as infants born with severe PIDs such as SCID, HSCT is a life-saving treatment. In recent years, HSCT is increasingly administered to young adults and for additional conditions, such as congenital neutropenia. According to the PID Principles of Care (2), HSCT should be available to all patients with relevant PIDs, independent of where they live. Despite this, there are many countries where this transplantation is not available for patients with PIDs but only for other diseases (2). According to this study, 40 countries stated that HSCT is available for patients with PIDs, compared to 15 countries that do not provide access to this treatment (**Figure 4**).

Another course of curative treatment is gene therapy, which involves inserting a working copy of the gene into the patients’ cells to supplement the gene that is defective or absent (2). Compared to HSCT, gene therapy offers treatment to patients who have no suitable donors (15). Gene therapy is currently only available in a few specialised centres for a small number of immune diseases. In this study, 10 of the participating countries reported having access to gene therapy (**Figure 4**). Among these only one country is located outside Europe (the US). As many as 44 countries reported that they do not have access to gene therapy. Even if it is predicted that more commercialised products will be made available in the coming decades (16), these therapies remain costly (for more details on reimbursement, please refer to the section on Universal Health Coverage) and require highly specialised teams and infrastructures that are, for the time being, only available in a small number of countries.

Thymic transplantation is, like gene therapy, also scarcely available to patients with PIDs as a therapeutic option, and currently only performed in two centres in the world (17). Out of 54 countries 10 have indicated that they have access to this treatment (**Figure 4**), although they will need to travel to these two centres to receive it. It is also important to note that 6 out of 54 respondents did not know whether this option was available to PID patients. Thymus transplant is a specialised treatment for very specific PIDs in which infants are born with no thymus (as in children born with complete Di George syndrome/athymia). As this therapy is currently still under investigation, it could explain the lack of awareness about its existence.

#### 3.2.4 Vaccines

Vaccines protect people against serious and life-threatening infectious diseases (18). Some patients with PIDs should not be given live-attenuated vaccines as they will not respond to them, and the attenuated virus may revert to virulence. In general terms, vaccines are available for patients with PIDs in most of the countries that have contributed to the Index. Only a few exceptions have said that almost all the vaccines are available, and few countries have selected “partially” as an answer. The WHO considers immunization as a “key component of primary health and an indisputable human right. It’s also one of the best health investments money can buy” (19). Many international and national efforts have been put into extending the vaccination coverage for many years and these efforts could well explain the wide availability of vaccines for patients with PIDs. Vaccination of the general population does not only protect those that are vaccinated but also reduces the impact on the most vulnerable groups that cannot receive the vaccines due to their immunocompromised system.

#### 3.2.5 Other Treatments for PIDs

**Figure 5:**
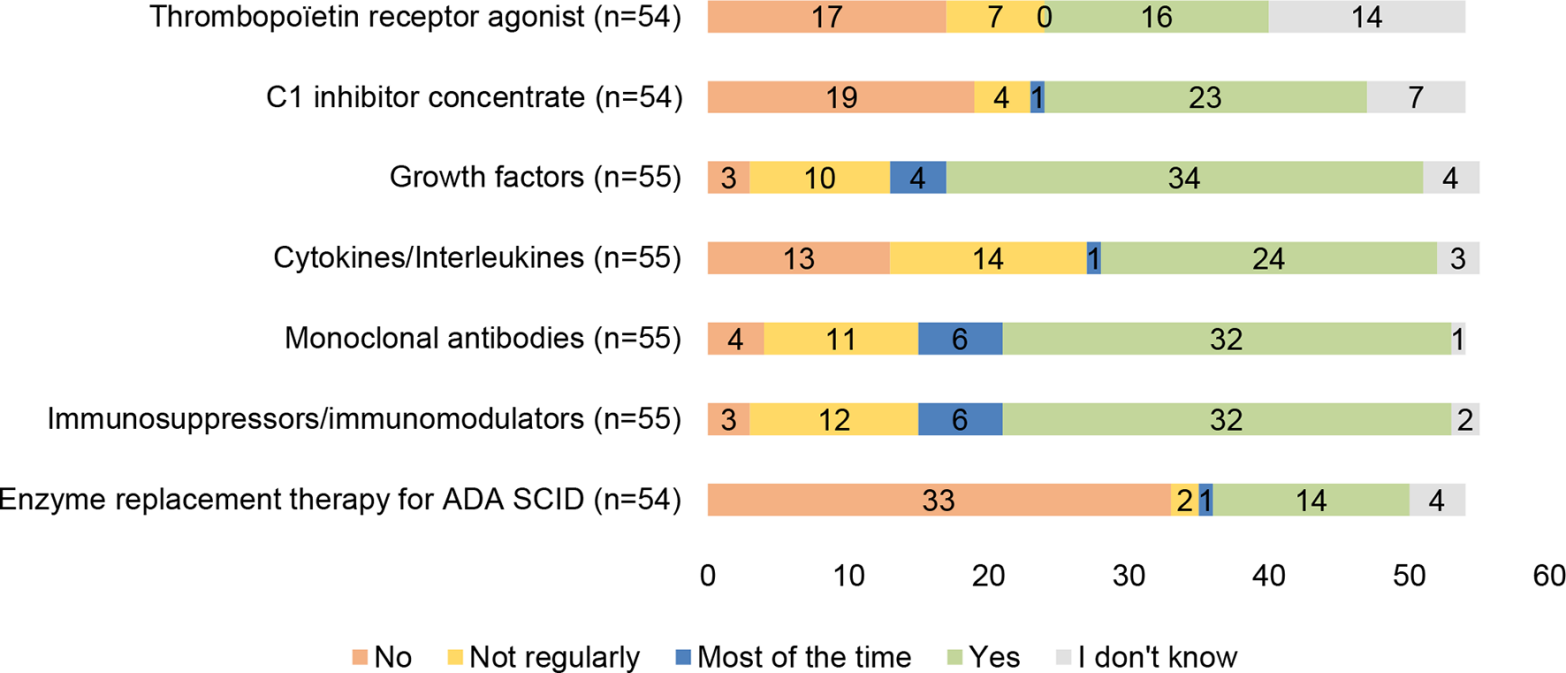
Availability of other treatments for patients with PIDs in individual countries.

Other therapies for the treatment of patients with PIDs are very heterogeneous, often referring to therapies that are required for very rare conditions. These include 7 main therapies: thrombopoietin receptor agonists, C1 inhibitor concentrate, growth factors (G-CSF, GM-CSF, EPO), cytokines/interleukins (Interleukin alpha, Interferon gamma, IL-2, IL-7, etc.), monoclonal antibodies, immunosuppressors and immunomodulators, and enzyme replacement therapy for ADA SCID. Their availability for patients with PIDs varies greatly between countries. In general terms, there is less knowledge of their availability compared with other more widely used therapies such as Ig therapies or anti-infectious treatments. Growth factors are the most widely available, present in 34 of the 55 countries that have responded to this question (**Figure 5**). On the other hand, enzyme replacement therapy for ADA SCID, used as a prophylactic therapy while the patient with ADA deficiency awaits HSCT or gene therapy, is the treatment that is less frequently available, with no availability in 33 of the 54 countries that have responded to the question.

### 3.3 Principle 3: Universal Health Coverage

Having a diagnostic method or a therapy available in a specific country does not mean, by default, that all patients with PID will have access to it without financial hardship. The WHO considers that “universal health coverage implies that all people have access without discrimination to nationally determined sets of the needed promotive, preventive, curative, palliative, and rehabilitative essential health services, and essential, safe, affordable, effective, and quality medicines and vaccines, while ensuring that the use of these services does not expose the users to financial hardship” (20). We may add that the coverage differs depending on the existence of a national health system in which children can be considered but not adults or is restricted to certain groups of the population (disabled people, civil servants, people serving in the army).

As the PID Life Index demonstrates, there are still many places in the world where PID care is a challenge in terms of affordability for patients or their families, especially in countries with scarce resources or in high competition with other endemic infectious diseases, such as tuberculosis, malaria, AIDS or chronic conditions such as cancer or diabetes.

**Map 1:**
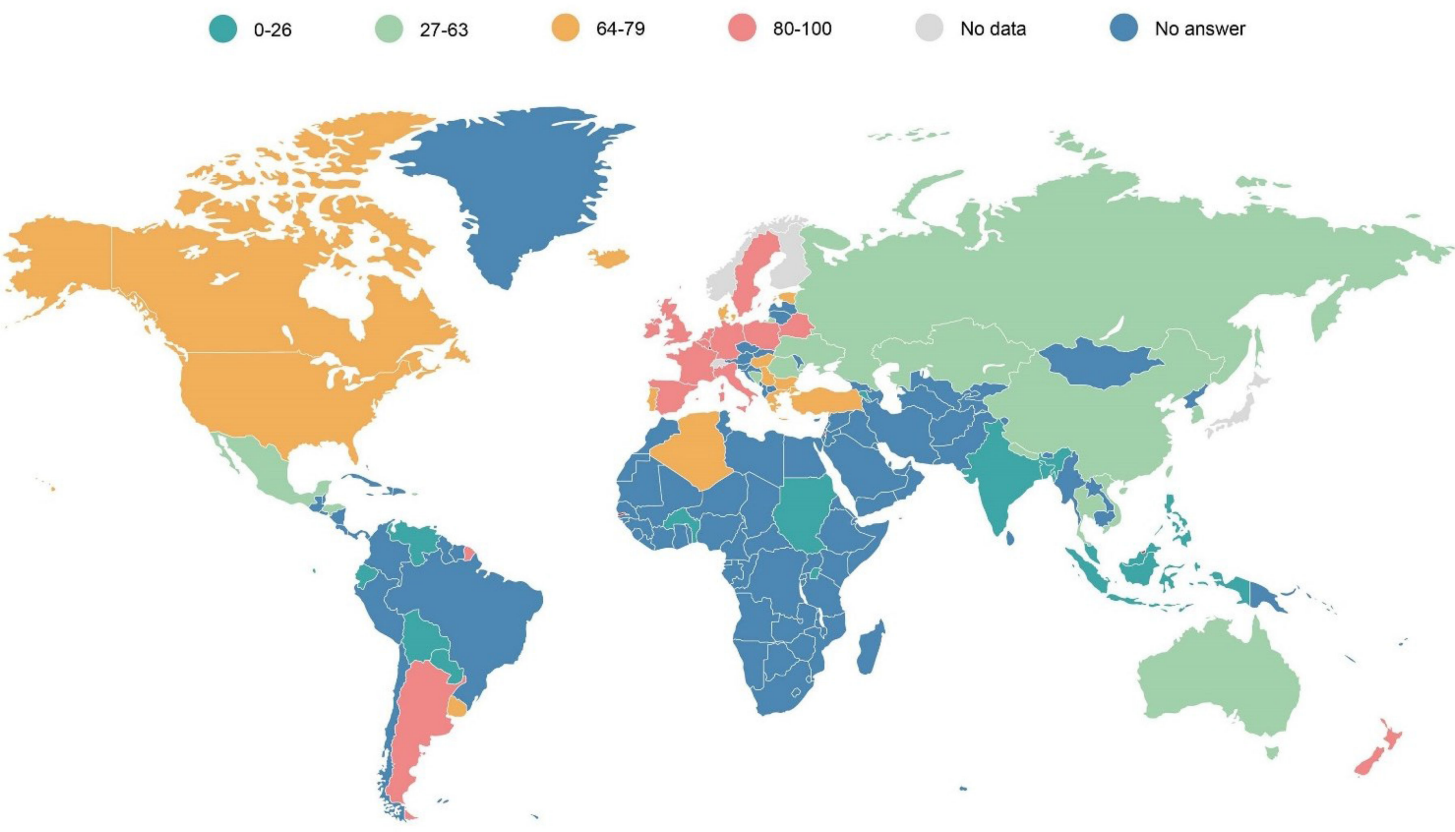
Universal health coverage of diagnosis and treatments for patients with PIDs (in % of the total cost).

Looking at the general overview of how diagnosis and treatment for patients with PIDs is covered by their national or regional systems, only a few countries, mainly located in Europe, provide health coverage of between 81 to 100% of the price of the diagnostic test or treatment to patients with PIDs (**Map 1**). There are still many countries in the world, mainly in Asia, Africa or Latin America, in which a very limited proportion (from 0 to 24% of the price) is covered for the diagnostics or treatment of patients with PIDs. This hampers access to the wider community of patients that do not have the financial means to purchase the diagnostic tests or therapies they need to enjoy their fundamental right to the highest attainable standard of health without distinction of, amongst others, their economic or social condition (18).

#### 3.3.1 Coverage of Reimbursement for Diagnostic Tests for PIDs

**Figure 6:**
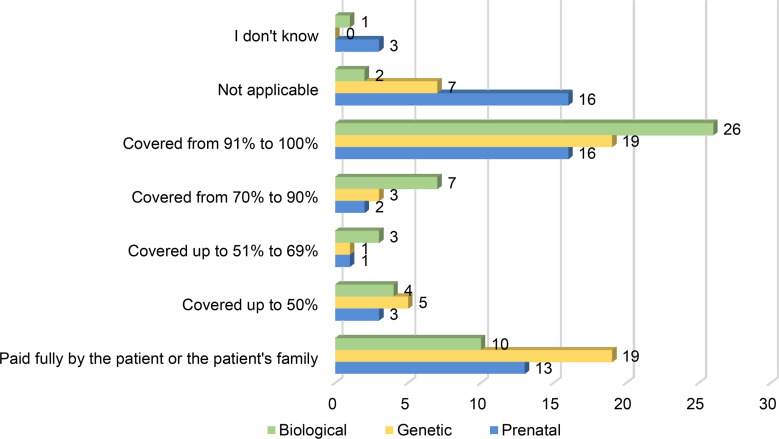
Coverage Levels of Diagnostic Tests (in % of the Total Costs) (n=53, 54, 54).

The reimbursement of diagnostic tests for PIDs varies greatly across the world and depends on the type of test. **Figure 6** shows the coverage levels of the three diagnostic tests the PID Life Index covers. Biological diagnosis is the type of test that is mostly covered fully by the health or social systems of the countries. Still, there are great disparities in access as in 10 countries, patients or their families need to cover the price of the test themselves, and in 4 countries less than half of the price is covered by the social system. This is the first very big barrier for patients to access a diagnosis.

Genetic diagnosis is the type of test that patients or their families often need to pay out of pocket. In 19 countries out of 54, patients need to pay for them fully and in 7 countries the tests are not even available. This means that in half of the countries surveyed, patients either do not have access to this type of test or need to fully pay for them, which is an impossible option for many patients. Prenatal diagnosis is the category of tests less covered in full by countries, as only 16 out of 54 include them in the national or regional reimbursement or coverage system.

#### 3.3.2 Coverage of Reimbursement for Anti-Infectious Therapies for Patients With PIDs

**Map 2:**
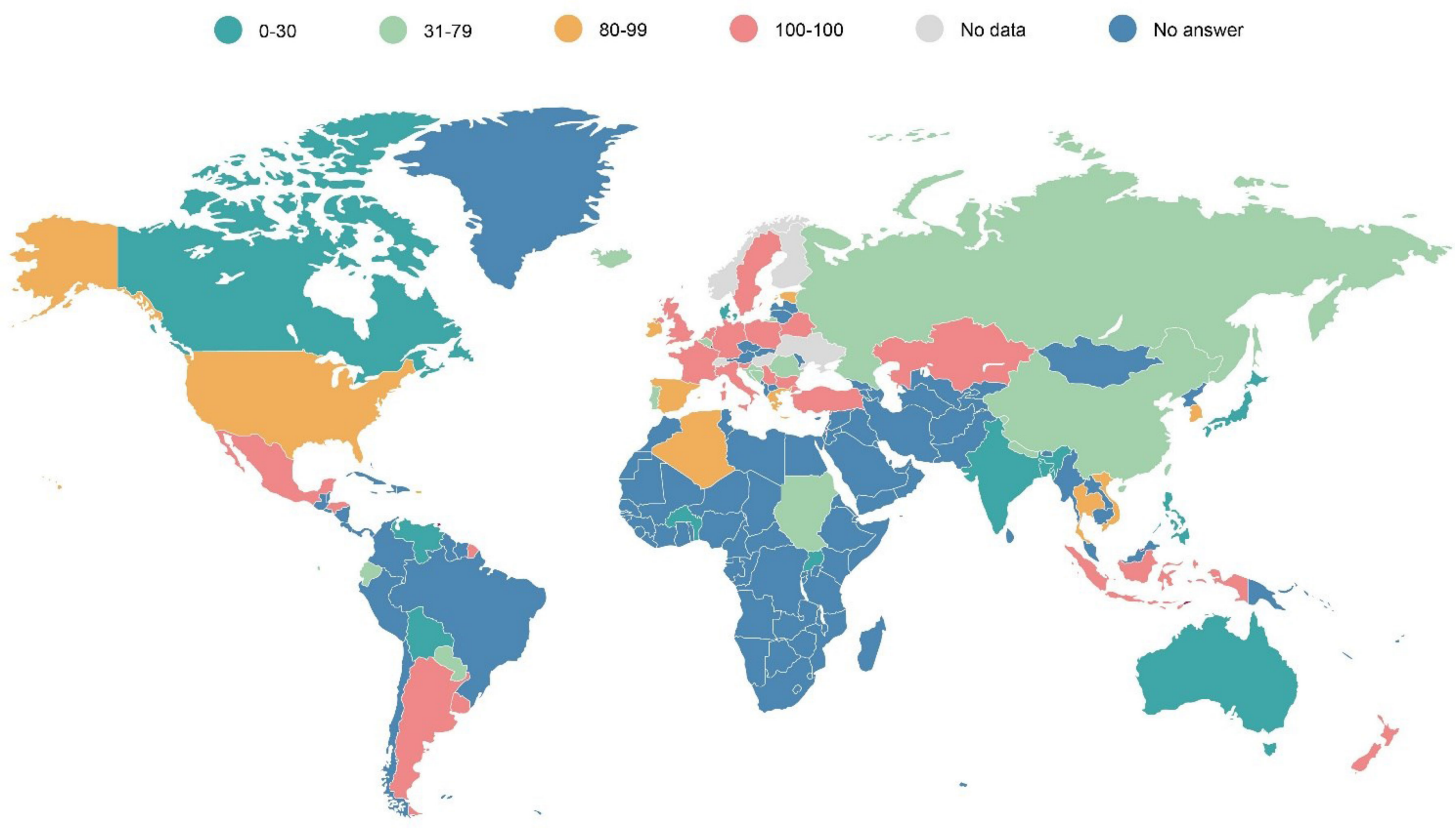
Coverage of anti-infectious therapies for patients with PIDs (in % of the total costs).

As demonstrated in the section dedicated to the availability of anti-infectious therapies, this type of therapy is available in most countries for patients with PIDs, with only a few exceptions. In terms of coverage by the health and/or social security services, anti-infectious therapies are widely covered or reimbursed (**Map 2**). Some interesting exceptions are Australia, Canada or Uganda, where anti-infectious therapies are available but only very partially covered in terms of price. Countries like India, Bolivia or Venezuela not only do not provide wide access to them, but they also do not cover them for patients with PIDs. Many countries may have chosen to only reimburse the most expensive therapies for chronic patients, such as patients with PID, leaving those therapies that are considered less expensive for the out-of-pocket expense by the patients.

#### 3.3.3 Coverage of Reimbursement for Ig Therapies for Patients With PIDs

Ig therapies are used by more than half of the patients with PIDs. They are considered as costly therapies in comparison to anti-infectious therapies and need to be taken for life by most patients, so having access to sustained coverage of the costs is a key issue for patients to be able to receive them as prescribed by their treating physician.

**Figure 7:**
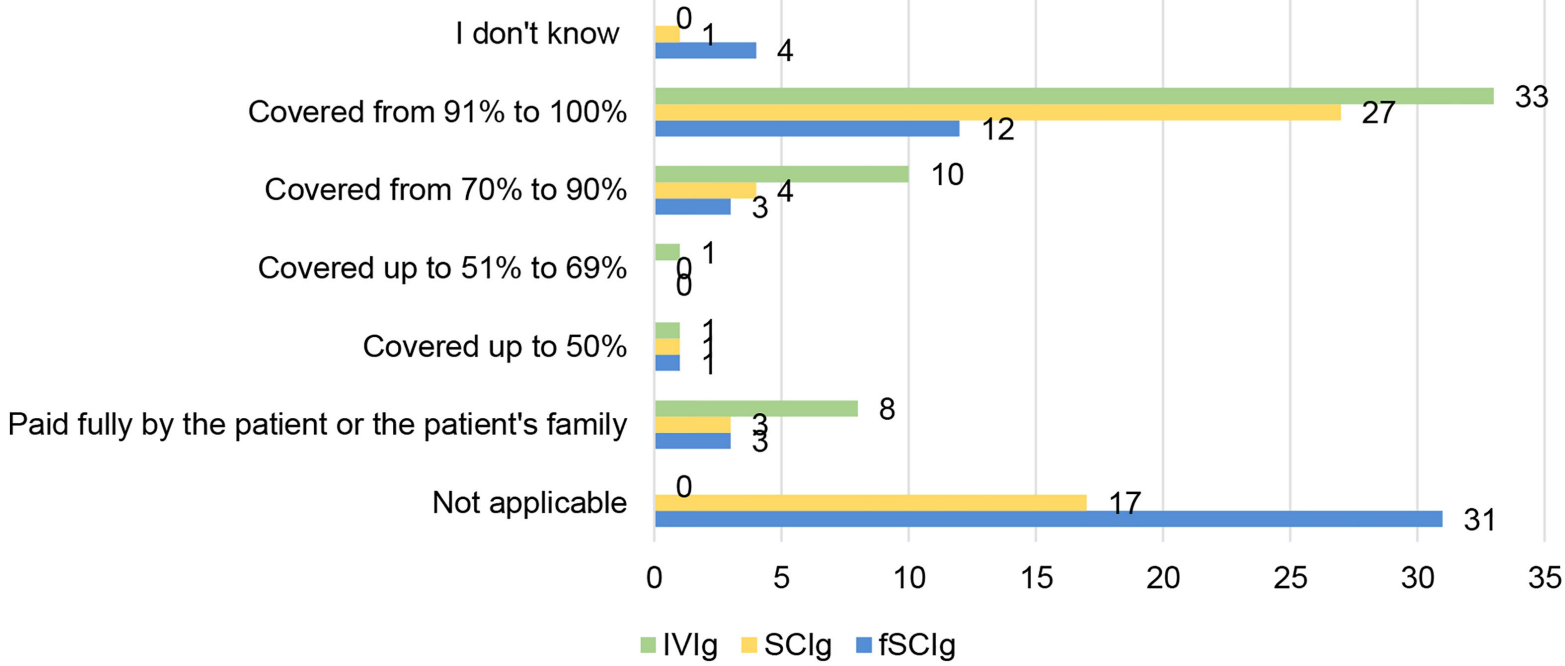
Reimbursement of Ig routes for patients with PIDs (in % of the costs) (n=53, n=53, n=54).

IVIg, the most frequent route of Ig in the world, is also the one mostly covered fully (from 91% to 100%): in 33 countries out of 53 (**Figure 7**). On the other hand, there are 8 countries out of 53 in which patients or their families still have to pay for the cost of the treatment themselves. For costly treatments such as Igs, this is a serious barrier to sustained access to therapies, showing the importance of advocacy of patient and physician organisations towards their governments to explain the importance of continued access to this type of therapies, the benefit this represents for the society to have these patients doing well and contributing not to speak of the need to ensure that no patient is left behind, no matter how rare their disease is.

SCIg is a treatment not available in 17 out of the 53 countries replying to this question (**Figure 7**). Half of the countries that have replied (27 out of 53) have reported full coverage for SCIg therapy. Looking at where the countries providing full reimbursement of SCIg are, they are located in Europe and northern America. In Latin America, Africa and Asia, only very few countries fully reimburse this route of administration: Argentina, Uruguay, Mexico or New Zealand. This shows the lack of access of patients with PIDs to a medicine considered essential by the WHO in many continents of the world.

Facilitated SCIg is a very recent therapy that in 31 out of 54 countries is not available and only covered fully in 12 out of 54 countries.

##### Coverage of Curative Therapies for Patients With PIDs

**Figure 8:**
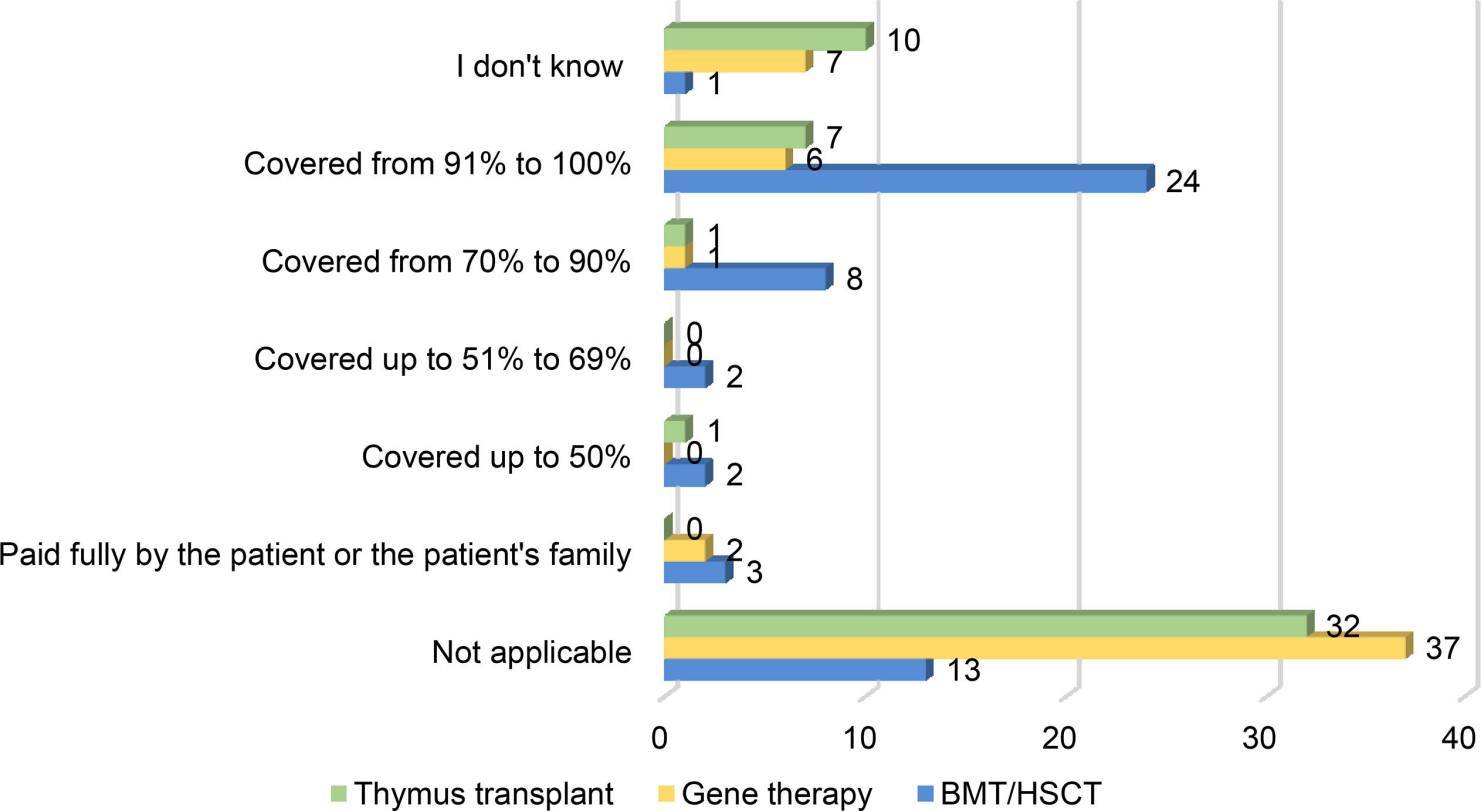
Coverage of the different curative treatments for patients with PIDs (in % of the costs) (BMT/HSCT n=53, Gene therapy n=53, Thymus transplant n=51).

When assessing the coverage status of each curative treatment in the PID Life Index, one can see an enormous difference between countries. In 13 out of 53 countries BMT/HSCT is not a possibility for patients with PIDs and in 3 countries it is either paid fully by the patient or the patient’s family (**Figure 8**). It is important to note that BMT/HSCT is a costly procedure that involves not only the medical procedure itself but also a set of arrangements such as travelling to and staying at the hospital for months, pharmaceutical costs, diagnostics costs (x-rays, MRI, clinical tests, etc), as well as indirect costs, such as absence from school or work, informal care by relatives (including relatives not being able to work to take care of the patient), etc (21). This particular procedure has been performed in patients with PIDs as a potential cure for the longest period of time, and this is reflected in the number of countries that provide them within their health benefits to their nationals. Nearly half of the countries (24 out of 53) offer the procedure within their coverage and 8 countries cover from 70% to 90% of the price. Although a larger dataset would offer further insight, the scope of this study did not allow for more in-depth investigation of the number of patients who needed BMT/HSCT and effectively received it without financial hardship. The available data instead provides a general coverage overview for BMT/HSCT, allowing for more focused research in future publications to offer more detailed insight on this principle.

Gene therapy is a procedure that is still relatively novel and has only received its first marketing authorisation for one type of PID in 2016. This is reflected in the coverage of the therapy in the different countries, with more than half of the countries not having it available (37 out of 53) and only 6 out of 53 offering full coverage (**Figure 8**). Looking at the geographical spread of these 6 countries, all of them are located in Europe.

Thymus transplantation is also not widely available (32 out of 51 countries) and only provided fully covered or free of charge in 7 countries (**Figure 8**). As in the case of gene therapy, these countries are all located in Europe. It is interesting to note that, although this type of procedure is a viable option for certain PIDs, the level of awareness is not as high as for the other curative therapies and 10 out of 51 country representatives have been unable to answer this question.

### 3.4 Principle 4: Specialised Centres

#### 3.4.1 National PID Specialised Centre/Network

People with PID should be managed in specialised centres to guarantee geographical access to medical and nursing expertise on their disease. The nature of these centres will vary depending on the available resources and expertise but should nonetheless reach internationally agreed standards of care and offer a holistic approach to diagnosis, treatment, and care of patients with PIDs. According to the criteria established in the PID Principles of Care, paediatric and adult centres (including transition care) should provide specialist diagnostic and management services, commit to training and professional development including PID research, ensure effective integrated care with other hospital specialities as well as have effective patient engagement (2 p. 3).

These centres should be connected in a network to guarantee regional accessibility, with formal links between the centres and recognised referral pathways for treatment and follow-up. The aim of such a network is to raise standards of care by sharing guidelines, registration, and peer review of PID centres, patient registries and professional leadership in PID. Access to a single coordinated network creates the optimal environment concerning appropriate and accurate diagnosis, treatment, and care for PID patients, independent of where they live.

**Figure 9:**
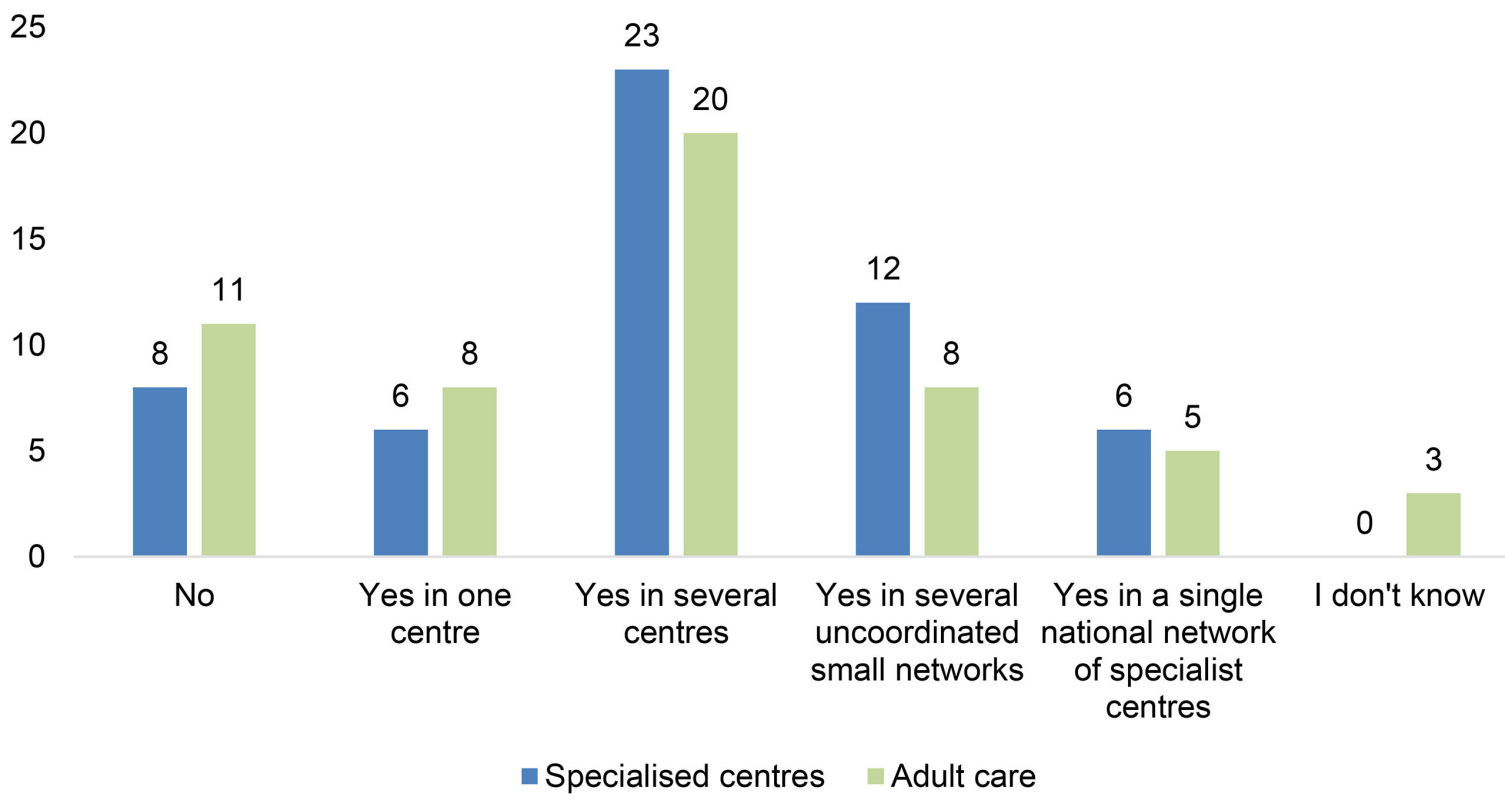
Availability of specialist and adult services for PIDs (n=55).

According to the PID Life Index, only 6 countries have specialist services available in a single coordinated national network (**Figure 9**), with all except for one country located in Europe. However, there are still many patients lacking access to an optimal diagnosis and treatment infrastructure. A significantly greater number of countries have access to specialist services in several independent centres (23 countries) or have services available in several uncoordinated small networks (12 countries). This is the reality for almost two-thirds of the countries in the PID Life Index. Although these countries do offer specialised services, what’s lacking is often a connection between independent centres or small coordinated networks. One single coordinated national network guarantees optimal care and equity, independent of the geographical location of the patient as well as offer optimal conditions for epidemiology and research on rare diseases.

In contrast, 14 countries indicated that patients only have access to specialist care in one centre or lack access altogether (**Figure 9**). In these countries, accessibility will remain a problem, especially for patients in remote areas. In countries with only one centre, patients may be confronted with the hurdles of long journeys to the national centre, impacting their social and professional lives as well as their ability to effectively attend all health checks and specialist visits. Lastly and most urgently, in countries where there are no specialised centres available, patients with PID run the risk of health implications and reduced quality of life and even life expectancy, increasing the healthcare burden not only on the patients but also on the healthcare provider/system due to unnecessary complications of their PID.

#### 3.4.2 Adult Care

Another important area to consider is the availability of adult PID healthcare services, offering a holistic approach to diagnosis and management of PID in adult patients. This concerns both patients who have transitioned from paediatric care as well as the large proportion of patients who have been diagnosed during adulthood. Many adult PID patients suffer for many years before being diagnosed and the number of undiagnosed adult patients is estimated to be high, due to lack of awareness. Still today, PIDs are often considered to be only paediatric diseases, especially in the developing world and although many PIDs are diagnosed in children, others are more commonly identified during adulthood and it is well established that PIDs affect both children and adults. One example of this is common variable immunodeficiency (CVID), which is the most common PID diagnosed in adult life (1 in 25 000 people, adult majority) (22, 23). Other examples of PIDs commonly identified in adults are immunoglobulin A deficiency and immunoglobulin G subclass deficiency.

According to the PID Life Index, adult care in a coordinated national network is only available in 5 of the 55 countries responding to this question (**Figure 9**), demonstrating a major shortcoming in the prioritisation of this area. In countries where it is available, the most common approach is to provide access in several uncoordinated centres (20 countries). The availability of several centres is promising, but to ensure that all patients have access to specialised care it is necessary to coordinate this in one network, pooling expertise to diagnose and treat adult patients effectively throughout the country. This is also important in countries where the most common organisation of adult care is through uncoordinated small networks or even in different medical specialities in which understanding of PIDs is sketchy or missing.

As many as 11 countries indicated that they do not have adult care at all, painting a complicated picture not only for adult patients but also for paediatric patients transitioning into adulthood. In these countries, it is vital to work to ensure that adult PID patients have the right to the same level of care as paediatric patients. With improved early diagnosis rates an adult patient with PID can receive appropriate treatment and reduce the risk of infections and other complications (24). Moreover, when PIDs are left undiagnosed or are misdiagnosed, chronic illness and disability take a heavy toll on healthcare resources. Appropriate diagnosis and treatment for adult patients is therefore not only an important priority to avoid unnecessary suffering, but also preferable out of a cost-effective perspective (25).

#### 3.4.3 Transition Care

Patients with PID should be cared for by a paediatrician specialised in immunology during their childhood in conjunction with an adult specialist, and then ideally by an adult PID specialist during their adulthood. As adolescents and young adults (AYAs) grow older, they should progress from depending on their parents/carers to become responsible for their own health and well-being and aware of the impact of their disease and treatment in their careers, families and society (26). Transition care is the transition period from the paediatric to the adult centre, where specialists and their teams prepare for this change together with the patient and their close contacts. It should be a planned process that addresses the medical, psychosocial, and educational needs of AYAs with PIDs as they move from child-centred to adult-oriented health care systems (27). Through this process the patients should be empowered to self-manage, supported by their multidisciplinary team. The age to start preparing the patient and the family for transition varies, in some countries it is recommended at a specific age, and in some it depends on the maturity of the patient. Many AYA PID patients are followed by the same health care team for many years and both the patient and their close contacts may fear the transition to adult care where the patient needs to learn to become autonomous and aware of adult risks. It is thus important for the multidisciplinary team to cooperate to make this experience positive and efficient for the patient and bridge the gap between paediatric and adult care.

**Figure 10:**
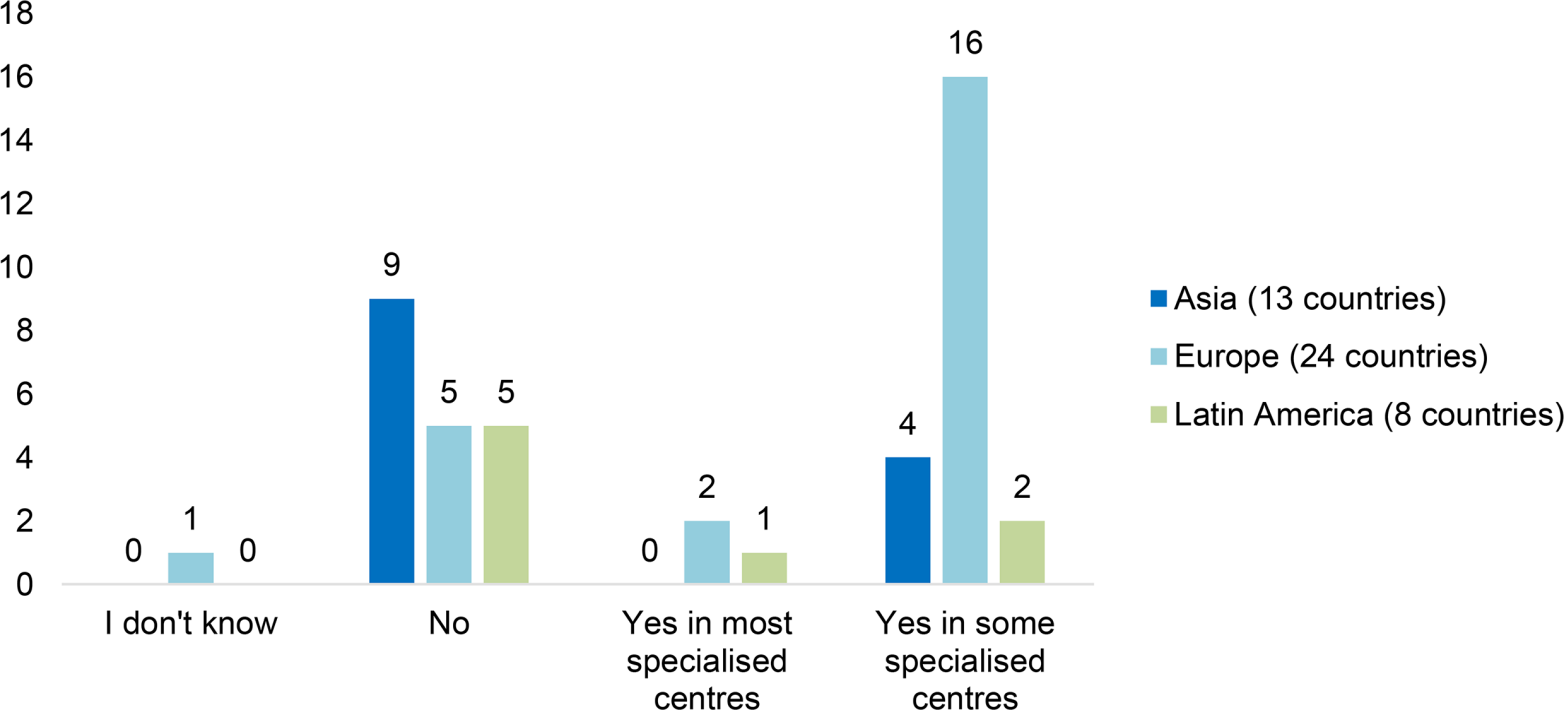
Availability of transition care specialist services for PIDs by region (n=45).

Based on data from the PID Life Index, there is still a vast amount of work needed to ensure that AYAs receive appropriate support and guidance during their transition. In countries where immunologists are not available or immunology is still considered a new speciality, transition care is still not widely implemented nor considered a priority. This is demonstrated by 22 of the answering countries where transition care from paediatric to adult services is not available at all. In 27 of these countries, transition care is implemented in some specialised centres, which should be contrasted with the mere 3 countries that indicated that it was available in most centres. Two of the regions where further measures to improve transition care is needed is Asia and Latin America, with 9 out of the 13 Asian countries indicating that they do not have access, and 5 out of 8 Latin American countries (**Figure 10**). IPOPI has a long history of working closely with doctors and patients to improve access to transition care, especially in Asia but also globally, however, continuous efforts are still needed to improve this principle.

### 3.5 Principle 5: National Patient Organisations

#### 3.5.1 Main Challenges

National Patient organisations for PID patients play a key role in health care systems and are becoming increasingly important stakeholders in the health care decision-making processes for rare diseases. Today it is well acknowledged that patient representatives have become specialists on their conditions and treatment and possess the unique ability to contribute with personal perspectives when advocating for their community (2 p. 5). Not only do they offer a supportive environment for patients and family members to exchange experiences and receive guidance, but many organisations also actively advocate to improve diagnosis, treatment and care for patients in their respective countries (28). The PID Principles of Care states that “All countries should aim to have an efficient national patient organisation, representing all PID patients – children and adults – in order to give them a voice and represent their interest in policy making” (2 p. 5).

Out of the 55 surveyed countries, 46 indicated that they have an established PID patient organisation and are members of IPOPI. In addition to this, there are more than 20 additional countries with national patient organisations (29), who are yet to contribute to the PID Life Index. The number of patient organisations varies between regions, and much work is needed until all PID patients have a national organisation to turn to. According to the PID Life Index, the region in the world that is in greatest need of development in this area is Africa, with only 2 countries contributing to the PID Life Index on this particular principle. The establishment of PID patient organisations could greatly improve the awareness of PIDs in this region, essential for improving diagnosis rates and ensuring appropriate treatment access for people with PID.

Some countries have newly established organisations, focusing mainly on patient support. In contrast, other organisations have been active in the field for decades and engage in a wider range of activities such as advocacy projects, awareness campaigns, research projects and more. Planning and participation in such activities require organisational capacity and expertise. To measure this capacity the PID Life Index investigated the number of full-time equivalent staff members in the organisations, ranging from no staff members to 5 full-time equivalent persons and above. As many as 36 out of the 46 responding countries operate without any staff members at all. In contrast, only the United States of America indicated that their national patient organisation has more than 5 full-time equivalent staff members. Between these two “extremes”, 7 countries are supported by 2-4 full-time equivalent staff members (Sweden, France, Germany, Russia, Canada, Australia, New Zealand), and 2 by 1 full-time equivalent staff member (Italy, United Kingdom). The growth and consolidation of PID patient organisations are essential to ensure that the interests of PID patients are represented. Securing enough expansion to allow for staff support will doubtlessly facilitate this task.

#### 3.5.2 Main Areas of Concern

**Figure 11:**
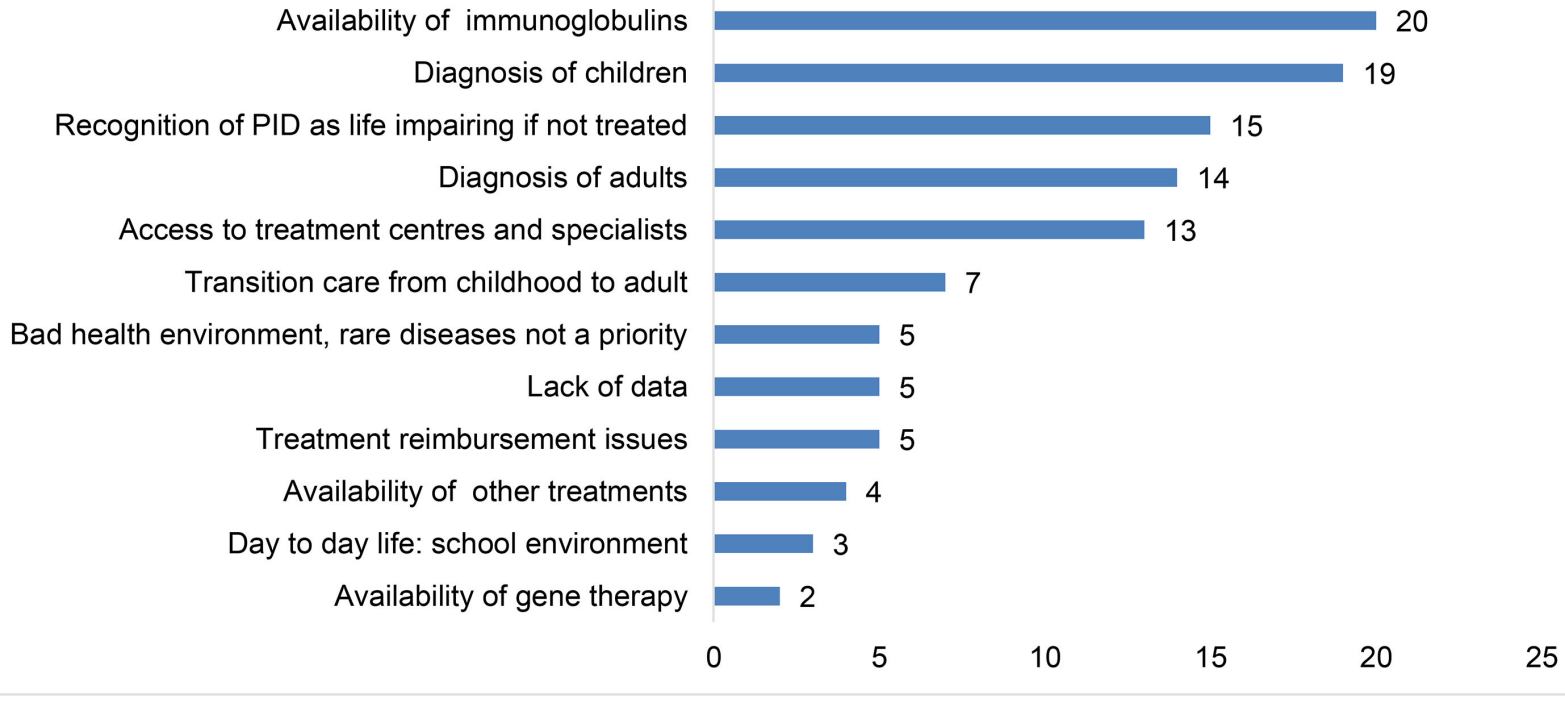
Main areas of concern for PID patient organisations (n=44).

PID patient organisations face different circumstances depending on their national setting, but many still struggle with similar challenges. One of the most common areas of concern is the availability of immunoglobulins, as indicated by 20 countries (**Figure 11**). As over 50% of affected PID patients suffer from antibody deficiencies (7) and replace the missing antibodies with immunoglobulin replacement therapy, this concern is not surprising. Stable and sustainable access to this lifelong treatment, with a variety of products and administration routes, is necessary to ensure good quality of life for many patients. Regrettably, there are still countries where this is not the reality. For PID patient organisations in developed countries, the main concern might be to ensure that patients have access to a wide range of products and administration routes, while in developing countries the main concern may instead be to ensure that at least one product is available. This demonstrates that circumstances may vary, but that continued and sufficient access to immunoglobulins nonetheless remains a real concern for PID patients. This concern also reflects the shortcomings regarding the levels of plasma collection needed to ensure global supply, with a strong geographical imbalance in the supply of plasma and growing demand for immunoglobulin treatment (30).

Another highlighted area of concern is the diagnosis of children (19 countries) and adults (15 countries) (**Figure 11**). Early diagnosis is critical for patients with PID to avoid unnecessary suffering and severe complications or even death. Although the diagnosis field is advancing rapidly, there are still many countries where this is not yet widely available, and where awareness of PIDs is low amongst general practitioners and paediatricians (31). In some countries, PID patients are only diagnosed after the loss of one, or even several older siblings, to the same disease. To avoid this unnecessary suffering and to ensure that patients have early access to appropriate treatment, improving diagnosis rates is a top priority for many patient organisations.

These national organisations also struggle with the lack of recognition of PID as a potentially life-impairing condition, impacting their education, employment as well as family and social life. For those without treatment, the impact may be severe. Without this recognition, people with PID may be subject to discrimination and find themselves without the possibility to benefit from the supporting legislation that an official recognition may bring. Impairment in interaction with societal barriers may hinder their full and effective participation in society on an equal basis with others (32), and this remains a real concern for many PID patient organisations.

#### 3.5.3 Main Working Areas

**Figure 12:**
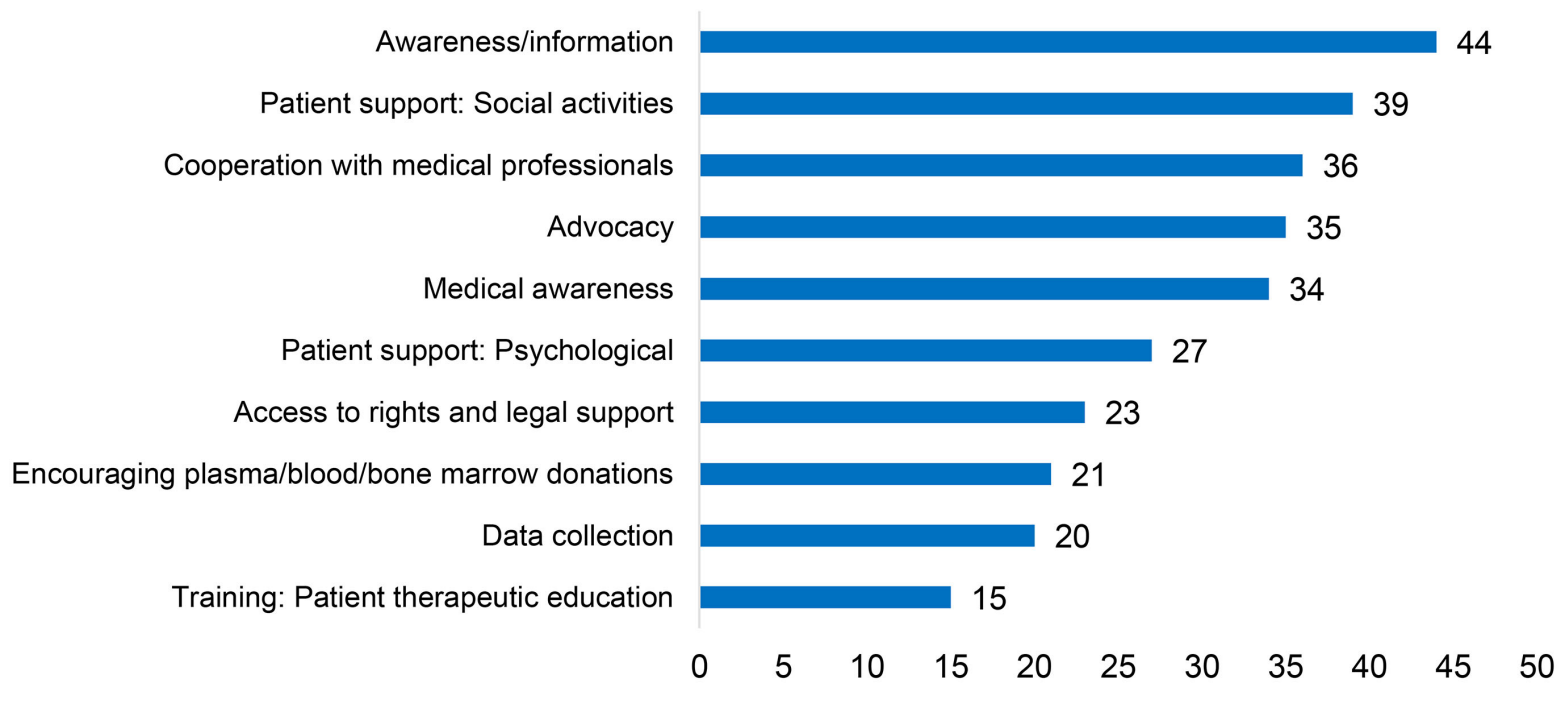
Main working areas for PID patient organisations (n=44).

One goal almost all the countries contributing to the PID Life Index have in common is that their PID patient organisations engage in awareness-raising activities (**Figure 12**), as a response to the challenges they face. Many patient organisations also disseminate important information to the medical community (33, 34) to reduce diagnostic delay, improve diagnostic rates and increase knowledge of these diseases. Another highly prioritised area concerns providing patient support, including psychological support, and organising social activities. This is a main working area for 39 out of the 44 countries. Beyond this, as many as 35 countries also engage in advocacy, with efforts to introduce newborn screening for SCID as a recent example and a growing number of patient organisations actively advocating for this cause. Patients with PID have everything to gain from advocating to ensure they are recognised and have access to the right diagnosis and timely treatment and care. As diagnostic tools and treatments exist, it is especially important to make policy makers or payers aware of this so that they can be made available and accessible.

### 3.6 Principle 6: National Registries

National registries are important epidemiological tools for assessing the proportion of affected individuals among the general population (prevalence), as well as for measuring the number of new cases diagnosed each year (incidence), detecting areas of low-diagnostic rates, providing insights on diagnostic delay associated with increased morbidity and mortality and studying specific populations or conditions (31). Beyond this, national and regional registries are recognised to be vital components of any public health programme, and provide data necessary for planning services, monitoring public health, research and the care of individuals (35). Not only is it necessary to collect national figures but given the scarcity of the individual types of PID, international databases are of importance to ensure that patient numbers needed for clinical research on PID is available. Currently, the availability and nature of these registries vary greatly. Some countries are equipped with up-to-date national registries (14 countries), while many lack registries altogether (38 countries). Some countries such as Malaysia (36), Vietnam and Algeria have registries based on data from a few medical centres, while others, such as South Korea, have a PID registry connected to only one medical centre. In contrast, in large countries with many medical centres treating PID patients such as Canada and the United States of America, finding ways to centralise the registries is a huge challenge that, if solved, could greatly benefit the PID community.

## 4 Discussion

The PID Life Index demonstrates that, regardless of global scientific progress with an increasing number of diagnostic tools and better treatment options for patients with PID becoming available, the accessibility and affordability remains uneven throughout the world. While some principles are becoming more widely implemented in some regions, others have much work ahead to reach full implementation. This discrepancy is not only visible between regions, but also comparing countries in the same region. The data shows that some countries do provide a better PID environment than others, but it also confirms that no country surveyed so far reaches a 100% score in the PID Life Index. Consequently, there is still nowhere in the world where the gold standard according to the PID Principles of Care is fully available and accessible to patients. Nevertheless, many principles or criteria are inter-linked and an improvement in one could imply an improvement in the other (e.g. existence of specialised centres in a country would likely improve diagnosis rates and allow for better treatment). It could thus be considered that countries channeling effort into implementing one principle may then improve other principles as well, and thus progress faster than expected.

The aim of this project has been to study the global PID environment through the IPOPI network. The invitation to participate was thus sent to IPOPI’s national member organisations (67 at the time), and to countries where IPOPI has well-respected medical contacts in the field of PIDs. Although this study offers a first of its kind presentation of key global data on PIDs, there are still PID centers that have not yet contributed to the index. Participation in this project was voluntary, and consequently a noticeable limitation is the lack of participation from several invited countries who decided not to take part or were not ready to provide the answers required. It should therefore be stressed that it is expected that, in the envisaged annual reviews of the data, new countries will contribute to the Index and ensure an even better understanding of the global PID environment.

### 4.1 Medical Education and Cooperation

A key theme visible when analysing the principles is the urgent need for medical education. In countries lacking immunologists, patients with PID risk being undiagnosed or misdiagnosed in other medical specialities and subject to health implications and even death. Educating the next generation physicians is key to disseminate immunologic scientific knowledge (37). So, improving medical education for PIDs allows for strengthened expertise and in turn to more accurate diagnosis and treatment for patients. In addition, increasing the number of immunologist clinicians allows for specialised centres to treat paediatric and adult patients, as well as the development of registries essential to accelerate research on PIDs. Ideally, the improved medical education should also be combined with international collaboration amongst medical societies and patient organisations, to ensure dissemination of knowledge to advance the PID environment globally. The existence of PID organisations and networks in many countries and most continents (e.g. ESID, LASID, ASID, APSID, ARAPID, USID-NET, CIS, SEAPID, ASCIA…) has helped to widen awareness of diagnosis and treatment for PIDs, with contributions from scientists, medical and nursing personnel as well as patient organisations; they should be encouraged to continue, in particular, to provide education of these rare diseases.

### 4.2 Lack of Infrastructure

According to the PID Life Index, two of the main concerns for many national PID patient organisations is poor access to diagnosis of children and adults (**Figure 11**). These concerns correspond well with the section on *Principle 1: Diagnosis of PIDs in the world*, where a low diagnosis rate was reported in the majority of the countries (**Figure 1**), confirming that PIDs are largely undiagnosed throughout the world (31). The concern of the patient organisations highlights a fundamental problem, namely the lack of infrastructure needed to carry out more advanced diagnostic tests including genetic testing and perform treatments such as HSCT or gene therapy. The incapacity to offer and cover these treatments is also a challenging barrier to newborn screening implementation, a screening test still only offered in a handful of countries at a national level. Once again, it is evident that the principles are interlinked and that improving one is likely to prove beneficial for the others.

### 4.3 Availability and Affordability

Having diagnosis and treatments both available and covered/reimbursed is key for patients with PID to access these in practice (3). In most countries, the necessary anti-infectious therapies are available and covered or reimbursed for patients with PID. In comparison, the full range of Ig therapies is only available in a few countries and in some countries, patients do not have access at all, or only on an irregular basis. As these biological medicines are not generic nor interchangeable (38), an individualised treatment plan and access to a wide range of products and administration routes are crucial for patients with PID. Despite the statement in the WHO Essential Medicines Lists where immunoglobulins are listed as essential therapies in the treatment of PIDs in adults and paediatric populations (9, 10), the data demonstrate that many countries still fail to make this treatment available and affordable in full range to patients. Another key point to consider is that although a treatment is officially considered available on a national level, geographical access may remain an issue for patients for which travelling far to receive treatment is not an option. This should also be taken into account when considering access issues.

Positively, most countries report having access to IVIg and it is also the one most covered for patients (**Figures 3**, **7**). However, in the 8 countries where patients need to pay out of pocket, actual accessibility can be contested as the high cost creates a real barrier for patients. Availability without affordability challenges WHO’s resolution on universal health coverage, where it is stated that all people should have access to essential health services, medicines, and vaccines without financial hardship (20). Only a few countries, mainly located in Europe, fully cover the costs for diagnosis and treatments for patients with PID. This remains a huge obstacle for access and it is something PID stakeholders need to continue to advocate to improve, to ensure a better quality of life for patients with PID.

### 4.4 Patient Centricity

The data manifests a vast landscape of national PID patient organisations engaging to improve the environment for PID patients in most of the surveyed countries. These organisations have a significant role to play in healthcare systems, bringing unique perspectives on the impact of diagnosis, treatment, and quality of life. It is therefore not only important to ensure patient-centred care for patients with PID for the well-being of the individual patient (i.e. individualised treatment plans), patient centricity and involvement are also key to reach a gold standard for PID from a more long-term perspective. Importantly, patient-centricity should not only be a symbolic word but put into practice and patient representatives should have a say in decisions and policies affecting their lives. Cooperation between patient organisations, other PID stakeholders and health decision-makers is thus of paramount importance to reach the common goal; a better national PID environment for patients in all countries.

## 5 Concluding Remarks

The implementation of the PID Principles of Care is progressing globally, but discrepancies remain between countries and regions. Main challenges are still clearly noticeable in the data, with a largely underdiagnosed patient population and poor access to appropriate treatment posing real hurdles to good quality of life for people with PID. Universal Health Coverage remains a challenge in many parts of the world, with treatments potentially being available but not yet affordable. Positively, many countries have access to specialised PID care in some centres, but coordination into one network is still not often considered. Moreover, in countries without access to transition or adult care patients with PID risk impaired health and possible comorbidities. Many patient organisations already advocate for their cause, but more collaboration between PID stakeholders is key to fully implement the principles and improve the quality of life for patients in all countries.

The PID Life Index provides an integrated worldwide view of the actual situation of PID Principles of Care, which conforms a cornerstone for what needs to be done next and a catalyst for creating new value for the patients. The data aggregated in the Index is the first of its kind for primary immunodeficiencies and offers a good basis for benchmarking, information sharing and for awareness and advocacy purposes. It handles a vast amount of data and offers easy access to essential principles and criteria for patients with PID, supporting the analysis of the current global PID environment and the way forward.

Limitations of this tool have been described elsewhere (article pending publication) and include: a nation-wide approach may not suit all countries, discrepancies between policy and how this policy is implemented, country coverage dependent on the existence of a PID patient group or a medical contact of IPOPI and the respondent’s personal bias depending on his/her knowledge and good faith. As this is a process, we consider that much of the bias will be reduced over time and the expansion of the number of countries responding will allow for the consolidation of the questionnaire and robustness of the data. This publication has allowed for a general presentation of the global status of the six principles, but a more in-depth analysis of each principle and how they correlate to each other is needed to fully grasp the status of each PID Principles of Care and what steps are inevitably needed to reach the gold standard.

## Data Availability Statement

The original contributions presented in the study are included in the article/**Supplementary Material**. Further inquiries can be directed to the corresponding author.

## Author Contributions

JN and LS wrote the manuscript and prepared and analysed the figures, supported by JP and MP. NM, HC, SS-R, AA, and JS contributed to the modification and revision of the manuscript. All authors contributed to the article and approved the submitted version.

## Funding

The development of the IPOPI PID Life Index was supported by Shire (now Takeda).

## Conflict of Interest

LS, JN, and JP work for and MP and JS are Board members of IPOPI. IPOPI has previously received an unrestricted grant from Shire (now Takeda) to support the development of the PID Life Index. IPOPI has also previously received unrestricted grants from Takeda and support from a broad range of companies involved in the field of primary immunodeficiencies outside the scope of the PID Life Index development. For an updated list of IPOPI’s corporate partners please visit www.ipopi.org.

The remaining authors declare that the research was conducted in the absence of any commercial or financial relationships that could be construed as a potential conflict of interest.

## Publisher’s Note

All claims expressed in this article are solely those of the authors and do not necessarily represent those of their affiliated organizations, or those of the publisher, the editors and the reviewers. Any product that may be evaluated in this article, or claim that may be made by its manufacturer, is not guaranteed or endorsed by the publisher.
