# Supplementary material for: The PID Principles of Care: Where Are We Now? A Global Status Report Based on the PID Life Index
Source: Front Immunol. 2021 Nov 18;12:780140. doi: 10.3389/fimmu.2021.780140 (PMC8637458; doi:10.3389/fimmu.2021.780140)
Supplement: Supplementary file 1 [file DataSheet_1.docx]

Supplementary Material

## APPENDIX 1:

| **Responding countries** |
| --- |
| Algeria, Argentina, Armenia, Australia, Bangladesh, Belarus, Belgium, Benin, Bolivia, Bosnia-Herzegovina, Bulgaria, Burkina Faso, Canada, China, Denmark, Ecuador, Estonia, Finland, France, Germany, Greece, Honduras, Hungary, Iceland, India, Indonesia, Ireland, Israel, Italy, Kazakhstan, Lebanon, Malaysia, Mexico, Nepal, New Zealand, Norway, Paraguay, Philippines, Poland, Portugal, Puerto Rico, Romania, Russia, Serbia, Slovenia, South Korea, Spain, Sudan, Sweden, Switzerland, Thailand, The Netherlands, Turkey, Uganda, Ukraine, United Kingdom, Uruguay, USA, Venezuela, Vietnam |
